# Unemployment and expenditure on health and education as mediators of the association between toothbrushing and global income inequalities

**DOI:** 10.1186/s12903-022-02570-y

**Published:** 2022-11-24

**Authors:** Maha El Tantawi, Nourhan M. Aly, Morenike Oluwatoyin Folayan

**Affiliations:** 1 Department of Pediatric Dentistry and Dental Public Health, Faculty of Dentistry, Alexandria University, Champollion St., Azarita, 21527 Alexandria, Egypt; 2 Department of Child Dental Health, Obafemi Awolowo University, Ile-Ife, Nigeria

**Keywords:** Toothbrushing, Gini coefficient, Income inequality, Unemployment, Policy

## Abstract

**Objective:**

The study assessed the association of country-level income inequalities with the percentage of schoolchildren toothbrushing-at-least-twice-daily; and the mediating effect of country-level unemployment rate and governmental expenditure on health and education (EH&E).

**Methods:**

This was an ecological study. The dependent variable was country-level toothbrushing-at-least-twice-daily among 11-15-year-old schoolchildren. Data for the period 2009 to 2019 were extracted from two global surveys about schoolchildren’s health and from manuscripts identified through a systematic search of three databases. The independent variable was country-level income inequalities measured by the Gini coefficient (GC) extracted from the Sustainable Development Report 2021. The mediators were the unemployment rate and EH&E. We stratified the sample by the level of GC and assessed the correlation between the dependent and independent variables in each stratum. Linear regression was used to assess the relations between the dependent and independent variables, and mediation path analysis was used to quantify the direct, indirect, and total effects.

**Results:**

Data were available for 127 countries. The mean (SD) percentage of children who brushed-at-least-twice-daily was 67.3 (16.1), the mean (SD) GC = 41.4 (8.2), unemployment rate = 7.5 (4.7) and EH&E = 8.4 (3.3). The percentage of children brushing at-least-twice-daily had weak and non-significant correlation with GC that was positive in countries with the least inequality and negative for countries with higher levels of inequality. A greater percentage of schoolchildren brushing-at-least-twice-daily was significantly associated with higher GC (B = 0.76, 95%CI: 0.33, 1.18), greater EH&E (B = 1.67, 95%CI: 0.69, 2.64) and lower unemployment rate (B=-1.03, 95%CI: -1.71, -0.35). GC had a significant direct positive effect (B = 0.76, 95%CI: 0.33, 1.18), a significant indirect negative effect through unemployment and EH&E (B=-0.47, 95%CI: -0.79, -0.24) and a non-significant total positive effect (B = 0.29, 95%CI: -0.09, 0.67) on the percentage of schoolchildren brushing-at-least-twice-daily.

**Conclusion:**

Unemployment and EH&E mediated the association between income inequality and toothbrushing. Country-level factors may indirectly impact toothbrushing.

## Introduction

Health behaviors influence the health and wellness of individuals. One of these behaviors is oral hygiene through toothbrushing. Infrequent toothbrushing is associated with greater caries incidence and increment in primary and permanent teeth [1]. Frequent toothbrushing reduces plaque accumulation and caries activity [2]. Less than twice daily toothbrushing at two and three years of age is associated with more caries at the age of 5 [3]. Toothbrushing also removes supragingival dental plaque which suppresses periodontopathogens in subgingival plaque and changes the microbial composition of the biofilm to one that is more compatible with periodontal health [4]. Toothbrushing, therefore, is a first line of defense against dental caries and periodontal disease; two oral diseases that affect large numbers of people worldwide [5].

Toothbrushing is associated with the socioeconomic status of the individual [6, 7] and the households [8]. Individuals with higher socioeconomic status clean their teeth more effectively and frequently and use more self-performed preventive strategies [9]. The socio-economic status may affect the ability to buy oral self-care products [10] although the financial investment required for this behavior is much lower than that needed for other more costly behaviors such as regular dental checkups [11]. Socioeconomic status may also affect access to oral health education that empowers one for safe and effective oral health care practices [10]. In addition, the stresses associated with having a low socioeconomic status reduce the ability to adopt a health behavior that require ongoing commitment such as toothbrushing [11]. Also, individuals with higher socioeconomic status may have higher levels of self-efficacy and inner locus of control in addition to greater likelihood of receiving social support and positive peer influence to adopt this behavior [11].

Measures of individual and household socioeconomic status correlate with Gross Domestic Product, income distribution (measured by the Gini coefficient of inequality), poverty and economic security [12].The association between income inequalities and worse health outcomes was demonstrated in case of oral health related quality of life [13], poor oral health [14], presence of caries and missing teeth [15], orthodontic treatment use [16], number of filled teeth [17], traumatic dental injuries [18], and lack of functional dentition among adults [19]. This association between oral health outcomes and income inequalities may be explained by inadequate investment in health and education and poor social cohesion [20] as well as higher prevalence of poverty, chronic stresses because of social comparisons and destabilization of the institutions safeguarding health [21]. Little is known, however, about the association between income inequalities and oral health behaviors such as toothbrushing.

Economists maintain that income inequalities impact growth and unemployment differently depending on country income level. Inequalities reduce innovation and growth and increase unemployment in poor countries [22, 23]. The negative impact of inequalities is due to less opportunities for workers and employers and thus, more likelihood of unemployment [23]. Inequalities affect low earning workers and they have a higher risk of employer exploitation, reduced productivity due to income insecurity, less purchasing power and consumer demand for goods and services. These factors further increase unemployment [24, 25].

On the other hand, inequalities may drive growth and reduce unemployment in rich countries by acting as incentive for the portion of society with less income to catch up and attain the same coveted positions as those with greater income. People with less income thus aim to create innovative products and services with more employment opportunities [26]. Also, greater income inequalities mean greater accumulation of financial capital in the hands of few persons to invest in projects creating more employment opportunities [27]. On the other hand, income inequalities are associated with less investment in education [27] which governments try to address by policies supporting the more vulnerable subgroups in the population. These policies aim to redistribute resources to balance out the accumulation of capital that occurs when inequalities increase and to reduce the cost born by individuals for education and healthcare. Thus, income inequalities affect employment and governmental expenditure on health and education.

A bulk of literature explains how to modify individual toothbrushing behavior through oral hygiene instructions, health education programs and behavior modification techniques [28–30]. However, country level determinants of toothbrushing need to be addressed through country-level policies and strategies. Research shows the value of adopting strategies to reduce the impact of income inequalities on health including increasing employment [31, 32] and promoting public investment in education to help individuals acquire skills needed for the job market [33–38]. These approaches are advocated by international associations [21] although it is not known whether they would reduce the impact of income inequalities on oral health behaviors such as toothbrushing. Understanding these interactions can contribute to the achievement of the resolution of the World Health Assembly by prioritizing oral health along with other non-communicable diseases [39]. The present study attempts to shed light on the role of country level income inequalities on oral health behaviors and the mitigating effect of spending on health and education as well as employment on this relation.

The aim of this study, therefore, was to assess the association between country-level income inequalities and the percentage of schoolchildren who brush their teeth, and whether this association was mediated by unemployment and governmental expenditure on health and education (EH&E). The hypothesis of the study was that greater income inequalities would be associated with lower percentage of children who brush their teeth; and that this association would be mitigated by higher employment rate and reduced by governmental measures to support population health and education.

## Methods

This was an ecological study that used data extracted from the databases of two global surveys and from literature review. The study included countries with nationally representative data about the percentage of schoolchildren aged 11 to 15 years who brush their teeth at-least-twice-daily from 2009 to 2019.

### Data sources

#### Dependent variable

##### Proportion of school aged children who brushed their teeth at-least-twice-daily

Data about the percentage of schoolchildren aged 11–15 years who brush their teeth at-least-twice-daily were obtained from the Health Behavior in School Children (HBSC) and the Global School-based Student Health Survey (GSHS). The HBSC is a school-based survey that collects data on health, well-being, social environments, and health behaviors of adolescents using a self-completed standardized questionnaire. The HBSC is conducted in collaboration with the World Health Organization (WHO) every 4 years since 1983. Initially, the HBSC collected data from European countries. Over time, the survey included countries outside Europe through the GSHS [40].

The HBSC and GSHS surveys used cluster sampling techniques where the classroom was the primary sampling unit [41]. The questionnaire was developed by an international research network, and administered to male and female schoolchildren aged 11, 13, and 15 years old. We focused on datasets of countries where toothbrushing was assessed. Data for one country were available per year from 2016 to 2019. Thus, the bulk of data was available in the six years from 2009 to 2015. From 2009 to 2015, data were available for a minimum of 3 and a maximum of 34 countries per year. In total, we extracted information from the HBSC and GSHS databases about 118 countries from 2009 to 2019. If more than one national survey was conducted per country, we used the most recent data [42, 43].

The extracted data for each country included responses to a question assessing the frequency of toothbrushing. The response options were: “more than once a day,” “once a day,” “less than once a day” and “never” which were recoded into “brushing-less-than-twice-daily” including “once a day,” “less than once a day” and “never” and “brushing-at-least-twice-daily” including “more than once a day”.

For the remaining 75 United Nations states without data from the two surveys, we conducted a systematic search to find studies or reports on the frequency of toothbrushing using the following criteria for document inclusion: (1) assessing the percentage of 11–15 year old children who brush their teeth, (2) reporting on toothbrushing-at-least-twice-daily or categorizing the frequency of toothbrushing to allow the calculation of this frequency, (3) using nationally representative sample or a sample from multiple and varied sites including the capital, urban and rural areas, (4) collecting data from 2009 to 2019.

We searched the following databases: MEDLINE, using the terms ((“Toothbrushing/ statistics and numerical data“[Mesh]) AND “Adolescent“[Mesh]) AND national AND “X“[Mesh]); Scopus, using the terms (toothbrushing AND frequency AND adolescents AND X); and Google Scholar, using the terms (toothbrushing AND adolescents AND national AND X) then (national AND dental AND survey AND X) where X was the country name. We checked the first 100 results in the Google Scholar search. The search was done independently by MET and NMA and differences were resolved by discussion.

#### Independent variable

##### Gini coefficient

Data about the Gini coefficient (GC) of income inequality per country were obtained from the database of the Sustainable Development Report 2021 [44]. The GC is an econometric indicator assessing inequalities in the distribution of wealth and income. The values of the coefficient range from zero if wealth is evenly distributed across people in the country and all persons have the same wealth, to 100 where wealth is concentrated in one person and thus, people have the greatest amount of differences [45]. The GC facilitates comparisons among groups because it has lower and upper bounds [46]. The GC has been previously used to quantify inequalities in health outcomes at national and subnational levels [47, 48] and at a global level [49]. The GC was used as a quantitative variable then further categorized into the following five categories: <0.2 indicating perfect income equality, 0.2–<0.3 indicating relative equality, 0.3–<0.4 indicating relatively reasonable income gap, 0.4–<0.5 indicating high income disparity, and ≥ 0.5 indicating severe income disparity [50].

##### Mediators

Two mediators were included: (1) unemployment rate calculated as the percentage of people in the labor force without work but available and seeking employment. The value of this indicator ranges from zero to 100 with greater values indicating more unemployment and, therefore, worse economic condition in the country, and (2) governmental expenditure on health and education which is calculated as the amount of this expenditure divided by the gross domestic product multiplied by 100, hereafter referred to as expenditure on health and education (EH&E). The values of this indicator, too, ranges from zero to 100 with greater values indicating greater governmental commitment to support the health and education of the population. Data about the two indicators were obtained from the database of the Sustainable Development Report 2021 [44].

##### Analysis

We calculated the percentage of schoolchildren brushing their teeth at-least-twice-daily for each country by dividing the number of children reporting at-least-twice-daily toothbrushing by the total number of children examined and multiplying by 100. We calculated the mean and SD for the percentage of schoolchildren brushing their teeth at-least-twice-daily, and for the independent variable and the two mediators. The correlation between the percentage of children brushing-at-least-twice-daily and the GC was assessed using Spearman rho per GC category.

SPSS was used to construct a linear regression model to assess the relations between the dependent and independent variable and the mediators and calculate standardized regression coefficients, unstandardized regression coefficients, 95% confidence intervals, and p values for each factor. We also added an Appendix with a table showing a regression model including additional variables with potential association with the dependent variable to explore whether the observed associations would be affected by adding other variables. We analyzed the mediation path using the PROCESS macro in SPSS, to calculate the direct, indirect and total effects of the GC on the percentage of children brushing their teeth at-least-twice-daily through EH&E and unemployment rate with double mediation. Mediation was considered to be present based on the joint significance test if both the effect of the independent variable on the mediators and the effect of the mediators on the dependent variable controlling for the independent variable were significant [34].

## Results

In addition to data about 118 countries from the HBSC and GSHS surveys, there were 76,13 search results for the remaining UN states. After excluding publications with non-relevant titles or abstracts, 56 documents remained. We further excluded publications if they had data before 2009 (*n* = 15), described a different brushing frequency (*n* = 13), included different age group (*n* = 9), or were based on non-national samples (*n* = 8). Eleven publications remained reporting on the percentage of 11-15-year-old children brushing their teeth at-least-twice-daily in 9 countries. Thus, data about toothbrushing were available for 127 countries.

**Fig. 1:**
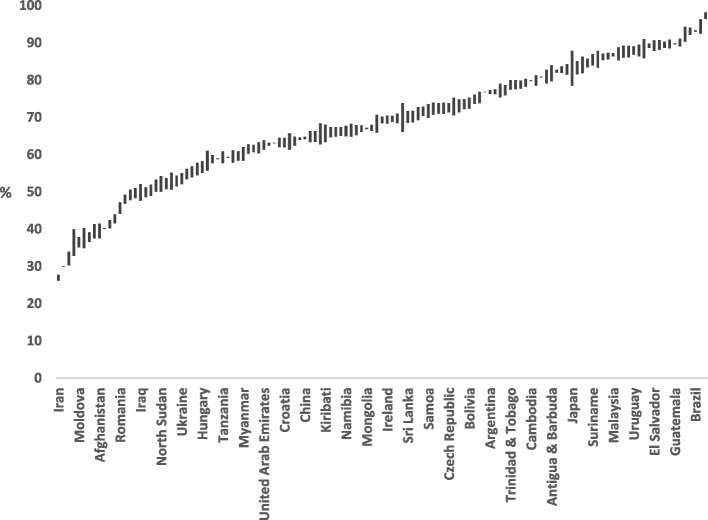
Percentage of children brushing-at-least-twice-daily in 127 countries (line for each country represents confidence interval of percentage)

Figure 1 shows that the percentage of children who brushed-at-least-twice-daily ranged from a minimum of 26.9% (95%CI: 26.2, 27.7) in Iran, 30% (95%CI: 29.9, 30.1) in Pakistan and 32.1% (95%CI: 30.2, 33.9) in Egypt, to 93.2% (95%CI: 93.0, 93.4) in Brazil, 94.7% (95%CI: 92.5, 96.3) in St. Lucia and 97.5 (95%CI: 96.4, 98.2) in St. Vincent and the Grenadines. There were 18 (14.2%) countries where less than 50% of children and 6 (4.7%) countries where more than 90% of children brushed-at-least-twice-daily. The mean (SD) percentage of children brushing-at-least-twice-daily was 67.26 (16.11). There was more than a 3-fold difference in the percentage of children with the highest and the lowest percentage of toothbrushing at-least-twice-daily.

The mean (SD) GC was 41.35% (8.18). The mean (SD) unemployment rate and EH&E were 7.51% (4.72) and 8.35% (3.33) respectively. Only 9.6% of countries showed relative equality, 32.7% had relatively reasonable income gap, 39.4% had high income disparity and 18.3% had severe income disparity. The correlation between the GC and the percentage of children brushing-at-least-twice-daily was weak and positive (rho = 0.04, *p* = 0.91) in countries with relative equality, weak and negative (rho= -0.20, *p* = 0.25) in countries with relatively reasonable income gap, weak and negative (rho= -0.08, *p* = 0.61) in countries with high income disparity and weak and negative (rho= -0.15, *p* = 0.54) in countries with severe income disparity.

Table 1 shows that the associations between the percentage of schoolchildren brushing-at-least-twice-daily and the GC (*p* = 0.001), the unemployment rate (*p* = 0.004) and the EH&E (*p* = 0.001) were statistically significant. The association with the GC was the strongest (β = 0.38) followed by the EH&E (β = 0.34) then the unemployment rate (β= -0.30). The associations with the GC and EH&E were direct: countries with 1% higher GC had 0.76 higher percentage of children who brushed-their-teeth-at-least-twice-daily; and countries with 1% higher EH&E had 1.67 greater percentage of schoolchildren who brushed-their-teeth-at-least-twice-daily. On the other hand, there was a negative association between brushing and unemployment rate where countries with 1% higher unemployment rate had 1.03 lower percentage of children who brushed-their-teeth-at-least-twice-daily. The associations between the percentage of schoolchildren brushing-at-least-twice-daily, net primary enrollment, lower secondary completion rate, literacy rate and gross national income per capita were assessed and they were not significantly associated (*p* = 0.51, 0.48, 0.57 and 0.79).

**Table 1:** The association between the percentage of schoolchildren brushing-at-least-twice-daily, GC, unemployment rate and EH&E in 127 countries

Variables	β	B (95%CI)	*p* value
Gini coefficient	0.38	0.76 (0.33, 1.18)	0.001
Unemployment rate	-0.30	-1.03 (-1.71, -0.35)	0.004
EH&E	0.34	1.67 (0.69, 2.64)	0.001

*β* Standardized coefficient, *B * Unstandardized coefficient, *CI * Confidence interval

**Fig. 2:**
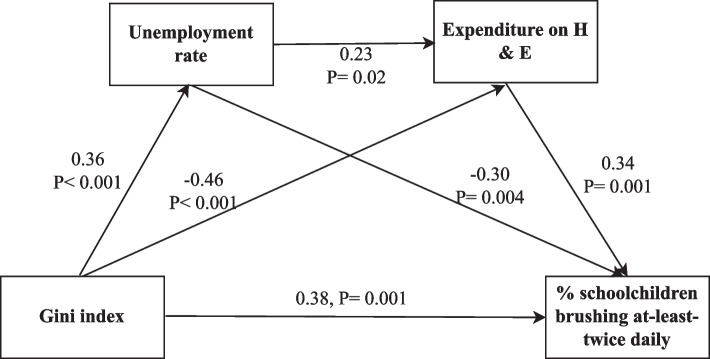
Mediation path analysis for the association between the GC and the percentage of schoolchildren brushing-at-least-twice-daily through unemployment rate and EH&E. Numbers are standardized coefficients and* p* values

Figure 2 shows that the GC was significantly associated with unemployment rate and EH&E (*p* < 0.001), and that the unemployment rate was significantly associated with EH&E (*p* = 0.02). EH&E, in turn, was significantly associated with the percentage of schoolchildren brushing-at-least-twice-daily (*p* = 0.001).

The joint significance test indicated that the association between the GC and toothbrushing was significantly mediated by unemployment and EH&E. Greater inequality was associated with greater unemployment (β: 0.36) and less EH&E (β: -0.46). Greater unemployment was associated with lower percentage of children who brush-at-least-twice-daily (β: -0.30). Also, greater EH&E was associated with a greater percentage of children brushing-at-least-twice-daily (β: 0.34).

Table 2 shows a significant positive direct effect (β: 0.38), a significant negative indirect effect (β: -0.24) and a non-significant total effect (β: 0.15) of the GC on the percentage of children who brush-at-least-twice-daily. There was no significant difference between the indirect effect of the GC on the percentage of children who brush-at-least-twice-daily mediated by the unemployment rate (β: -0.11) or by EH&E (β: -0.16) where the difference between the two mediated effects was 0.05 (95%CI: -0.07, 0.21).

**Table 2:** Direct, indirect and total effects for the association between the GC and the percentage of schoolchildren brushing-at-least-twice-daily

Effects	β	B (95% CI)	*p* value
Total	0.15	0.29 (-0.09, 0.67)	0.14
Direct	0.38	0.76 (0.33, 1.18)	0.001
All indirect	-0.24	-0.47 (-0.79, -0.24)	0.001
Indirect through unemployment rate	-0.11	-0.22 (-0.43, -0.066)	0.004
Indirect through EH&E	-0.16	-0.31 (-0.63, -0.11)	0.001

*β* Standardized coefficient, *B * Unstandardized coefficient, *CI *Confidence interval

## Discussion

The findings show that there were large variations among 127 countries in the percentage of 11-to-15-year-old children brushing-at-least-twice-daily and that about two of three children, on average, brushed-their-teeth-at-least-twice-daily. Overall, the greater the income inequalities, the greater the percentage of schoolchildren who brushed-their-teeth-at-least-twice-daily although splitting by level of inequality suggested possible differences in the direction of correlation. Unemployment and EH&E significantly mitigated the association between income inequalities and toothbrushing by reducing the significant direct positive effect to a weaker effect that was still positive but not significant. The findings, thus, partly support the study hypotheses and suggest that governmental policies affecting unemployment and EH&E may be associated with a weaker relation between income inequalities and frequent toothbrushing.

The study had some limitations. First, we used an ecological design where countries were the units of observation and analysis. This design is liable to ecological fallacy [51] where findings at country-level are generalized to individuals. However, the fallacy becomes a concern only if individual-level results refute the findings obtained at country-level. Ecological studies are useful in identifying differences among countries caused by macro level factors such as policies and income inequalities that should be further explored in other studies. Second, though we collected toothbrushing data from 2009 to 2019 for 127 (65.8%) of the 193 United Nations States, we could not find data for the remaining UN states. Despite this limitation, our study provides the most updated and comprehensive information about toothbrushing among schoolchildren worldwide, the largest number of countries in one study. Also, we attempted to minimize variation by time of data sources by extracting data about the exposure and mediators from the same source; the Sustainable Development Report 2021 [44]. Third, we considered only one independent factor and two mediators in the present study. We acknowledge that toothbrushing is a complex behavior that is associated with several determinants including oral health literacy and others. Not all of these factors were included in this analysis, and they need to be addressed in future studies. These factors might have been responsible for residual confounding that would explain the findings to unknown extent. Examples of these potential factors include expenditure on social security, political systems and health literacy. We explored the effect of adding some of these factors such as the literacy rate and Gross National Income per Capita (GNI) in the Appendix and the impact of other factors needs to be also assessed in future studies. Fourth, because of the limitation imposed by the number of countries with available data, it was not possible to conduct mediation analysis for groups of countries based on the level of income inequalities which might have shed some light on the modifying effect of levels of inequality. Fifth, data about toothbrushing was based on self-reporting which carries the risk of social desirability bias [52] that may differ from one country to another depending on culture [53]. However, self-reported toothbrushing is considered a suitable method for epidemiologic surveys [54]. Overall, the study has several important findings.

First, income inequalities were directly associated with a greater percentage of children brushing their teeth. This association disagrees with previous studies reporting an association between income inequalities and negative oral health outcomes such as indicators of oral diseases including caries experience in adults [55], and children [15], and tooth loss in adults [56]. However, a prior report showed that income inequalities were associated with better health outcomes measured by mortality describing what was called the Swiss paradox [20]. Similar to our situation, there was no clear explanation for the Swiss paradox. However, we observed a positive correlation between income inequalities and toothbrushing only in countries with the lowest level of income inequalities and negative correlation in countries with greater levels of inequalities suggesting a modification for the association between income inequality and toothbrushing by inequality level. This finding seems to agree with a previous Chinese study showing that the relation between health status and GC assumes an inverted U shape [57]; and with a meta-analysis indicating the presence of a GC threshold above which the association between income inequality and mortality differed from below it [58]. It is also possible that the observed association between income inequalities and toothbrushing in the present study is related to income level [59, 60] which needs to be investigated further.

Second, we observed that income inequalities had a direct positive effect on toothbrushing, an indirect negative effect and a positive total effect that was weaker than the direct effect. In mediation analysis, the direct effect usually has a smaller effect than the total effect because part of the association is explained by the mediators which are included in the calculation of the direct effect. In the present study, however, because the indirect effect was negative, the mediators acted as suppressors [61] causing the total effect to be less than the direct effect. Our study finding suggests that governmental interventions supporting the health and education of the population through public spending may indirectly reduce the impact of economic problems on toothbrushing by improving individuals’ access to education, employment and other socioeconomic factors. The present finding of how country-level policies may impact the association between income inequalities and toothbrushing also agrees with existing evidence that economic policies may affect health even if they are not aimed to do so [62].The study, thus, sheds light on how social policies may impact oral health through promoting toothbrushing.

Finally, the study also shows the complexity of the interrelationship between the macro-level determinants of toothbrushing and how they may interrelate to produce different effects on oral health behaviors. Toothbrushing is a core focus of oral health education programs for individuals, households and communities. Ignoring the country level contextual effect on toothbrushing frequency may overestimate the potential effectiveness of the education programs targeting individuals and the community and create unrealistic expectations.

Successful public health interventions link policies with activities that reduce the individual risks of non-communicable diseases such as tobacco cessation, promotion of physical activity, sugar restriction and promotion of healthy diets [63]. The study findings are important at the time of the call to embed oral health within the global non-communicable diseases control agenda and incorporate oral healthcare into universal health coverage [39]. The findings, also, suggest that countries may need to holistically assess the interactions of health and non-health policies and the possible impact they may make on oral health behaviors such as toothbrushing.

## Conclusion

Income inequalities were directly associated with frequent toothbrushing among 11- to 15-year-old children in 127 countries; and the correlation between them differed by the level of income inequality. Policies affecting unemployment and supporting health and education mediated the association between income inequalities and toothbrushing and reduced the direct effect. The interaction between economic conditions and policies on one hand, and between policies and toothbrushing on the other hand sheds light on the impact of country-level factors on toothbrushing and the importance of a holistic approach to address oral health issues.

## Data Availability

The datasets analyzed in the current study are available at the World Health Organization website for the Global school-based student health survey (GSHS) (https://www.who.int/teams/noncommunicable-diseases/surveillance/data) and the Health Behaviour in School-aged Children (HBSC) survey (https://gateway.euro.who.int/en/datasets/hbsc/).

## Appendix

Table 3

**Table 3:** The association between the percentage of schoolchildren brushing-at-least-twice-daily, GC, unemployment rate, EH&E, net primary enrollment, lower secondary completion rate, literacy rate and gross national income per capita

Variables	β	B (95%CI)	*p* value
Gini coefficient	0.43	0.90 (0.40, 1.41)	0.001
Unemployment rate	-0.41	-1.33 (-2.14, -0.51)	0.002
EH&E	0.30	1.96 (0.17, 3.76)	0.03
Net primary enrollment (%)	-0.09	-0.24 (-0.98, 0.49)	0.51
Lower secondary completion rate (%)	-0.11	-0.09 (-0.33, 0.16)	0.48
Literacy rate (% of population aged 15 to 24)	0.09	0.14 (-0.36, 0.64)	0.57
Gross national income per capita	-0.04	-0.00005 (-0.0004, 0.0003)	0.79

*β* Standardized coefficient, *B* Unstandardized coefficient, *CI *Confidence interval

Net primary enrollment, lower secondary completion rate, literacy rate are three indicators describing the literacy and education level at country level. They were extracted from the Sustainable Development Report 2021

Gross national income per capita is an indicator of country level economic level in 2021and was extracted from the World Bank Databank (https://datacatalog.worldbank.org/search/dataset/0038128/GNI-per-capita-ranking--Atlas-method-and-PPP-based)

## References

[1] Kumar S, Tadakamadla J, Johnson NW (2016). Effect of Toothbrushing Frequency on Incidence and Increment of Dental Caries: A Systematic Review and Meta-Analysis. J Dent Res.

[2] Goldenfum GM, Silva NC, Almeida IDA, Neves M, e Silva BB, Jardim JJ (2021). Efficacy of 1.23% acidulated phosphate fluoride gel on non-cavitated enamel lesions: a randomized clinical trial. Braz Oral Res.

[3] Boustedt K, Dahlgren J, Twetman S, Roswall J (2020). Tooth brushing habits and prevalence of early childhood caries: a prospective cohort study. Eur Arch Paediatr Dent.

[4] DeSpain Eden B. Prevention Strategies for Periodontal Diseases. In: Prevention in Clinical Oral Health Care. Mosby, Elsevier Health Sciences: Amsterdam, The Netherlands; 2008. pp. 213–29.

[5] James SL, Abate D, Abate KH, Abay SM, Abbafati C, Abbasi N (2018). Global, regional, and national incidence, prevalence, and years lived with disability for 354 diseases and injuries for 195 countries and territories, 1990–2017: a systematic analysis for the Global Burden of Disease Study 2017. Lancet (London, England)..

[6] Park JB, Han K, Park YG, Ko Y (2016). Association between socioeconomic status and oral health behaviors: The 2008–2010 Korea national health and nutrition examination survey. Exp Ther Med.

[7] Trinh VA, Tarbit E, Do L, Ha D, Tadakamadla SK (2021). The influence of family socioeconomic status on toothbrushing practices in Australian children. J Public Health Dent.

[8] Soofi M, Pasdar Y, Karami Matin B, Hamzeh B, Rezaei S, Kazemi Karyani A (2020). Socioeconomic-related inequalities in oral hygiene behaviors: a cross-sectional analysis of the PERSIAN cohort study. BMC Oral Health..

[9] Watt RG (2012). Social determinants of oral health inequalities: implications for action. Community Dent Oral Epidemiol.

[10] Costa SM, Martins CC, Bonfim M, de Zina LC, Paiva LG, Pordeus SM (2012). A Systematic Review of Socioeconomic Indicators and Dental Caries in Adults. Int J Environ Res Public Health.

[11] Pampel FC, Krueger PM, Denney JT (2010). Socioeconomic Disparities in Health Behaviors. Annu Rev Sociol.

[12] Osberg L, Sharpe A. Comparisons of Trends in GDP and Economic Well-being-the Impact of Social Capital Comparisons of Trends in GDP and Economic Well-being-the Impact of Social Capital 2. 2001. https://www.oecd.org/innovation/research/1824740.pdf. Accessed 1 Aug 2022.

[13] Alwadi MAM, Vettore MV (2019). Contextual income inequality and adolescents’ oral-health-related quality of life: A multi-level analysis. Int Dent J.

[14] Moeller J, Starkel R, Quiñonez C, Vujicic M (2017). Income inequality in the United States and its potential effect on oral health. J Am Dent Assoc.

[15] Celeste RK, Nadanovsky P, Ponce de Leon A, Fritzell J (2009). The individual and contextual pathways between oral health and income inequality in Brazilian adolescents and adults. Soc Sci Med.

[16] Simon L, Choi SE, Ticku S, Fox K, Barrow J, Palmer N (2020). Association of income inequality with orthodontic treatment use. J Am Dent Assoc.

[17] Campus G, Cocco F, Strohmenger L, Cagetti MG (2020). Caries severity and socioeconomic inequalities in a nationwide setting: data from the Italian National pathfinder in 12-years children. Sci Rep..

[18] Vettore MV, Efhima S, Machuca C, de Lamarca G (2017). Income inequality and traumatic dental injuries in 12-year-old children: A multilevel analysis. Dent Traumatol.

[19] Goulart MDA, Vettore MV (2016). Is the relative increase in income inequality related to tooth loss in middle-aged adults?. J Public Health Dent.

[20] Cohen SA, Greaney ML, Klassen AC (2019). A “Swiss paradox” in the United States? Level of spatial aggregation changes the association between income inequality and morbidity for older Americans. Int J Health Geogr..

[21] American Public Health Association. Reducing Income Inequality to Advance Health. 2017. https://www.apha.org/policies-and-advocacy/public-health-policy-statements/policy-database/2018/01/18/reducing-income-inequality-to-advance-health. Accessed 8 Oct 2022.

[22] National Bureau of Economic Research. Inequality and Growth. 1999. https://www.nber.org/digest/aug99/inequality-and-growth. Accessed 28 Oct 2022.

[23] Topuz SG (2022). The Relationship Between Income Inequality and Economic Growth. Are Transmission Channels Effective? Soc Indic Res.

[24] Cingano F. Trends in Income Inequality and its Impact on Economic Growth. 2014. https://www.oecd.org/els/soc/trends-in-income-inequality-and-its-impact-on-economic-growth-sem-wp163.pdf. Accessed 28 Oct 2022.

[25] AAPR International. What does rising income inequality mean for the future of work? 2022. https://www.aarpinternational.org/initiatives/future-of-work/megatrends/income-inequality. Accessed 28 Oct 2022.

[26] Birdsong N. The Consequences of Economic Inequality. 2015. https://sevenpillarsinstitute.org/consequences-economic-inequality/. Accessed 28 Oct 2022.

[27] Lahouij H. The Effects of Income inequality on Economic Growth Evidence from MENA Countries. 2017 Awards for Excellence in Student Research and Creative Activity – Documents. 4. 2017. https://core.ac.uk/download/pdf/154553817.pdf. Accessed 28 Oct 2022.

[28] Damle SG, Patil A, Jain S, Damle D, Chopal N (2014). Effectiveness of supervised toothbrushing and oral health education in improving oral hygiene status and practices of urban and rural school children: A comparative study. J Int Soc Prev Community Dent.

[29] Subedi K, Shrestha A, Bhagat T, Baral D (2021). Effectiveness of oral health education intervention among 12-15-year-old school children in Dharan, Nepal: a randomized controlled trial. BMC Oral Health.

[30] Almabadi ES, Bauman A, Akhter R, Gugusheff J, Van Buskirk J, Sankey M (2021). The Effect of a Personalized Oral Health Education Program on Periodontal Health in an At-Risk Population: A Randomized Controlled Trial. Int J Environ Res Public Health..

[31] Atkinson AB, Inequality (2015). What Can Be Done?.

[32] McNichol E. It’s time for states to invest in infrastructure. 2019. http://www.cbpp.org/sites/default/files/atoms/files/2-23-16sfp.pdf. Accessed 7 Oct 2022.

[33] Karoly LA, Kilburn R, Cannon JS. Proven benefits of early childhood interventions. 2005. http://www.rand.org/pubs/research_briefs/RB9145.html. Accessed 7 Oct 2022.

[34] Bezruchka S (2012). The hurrider I go the behinder I get: the deteriorating international ranking of U.S. health status. Annu Rev Public Health.

[35] Organisation for Economic Co-operation and Development. Reducing income inequality while boosting economic growth: can it be done? 2012. https://www.oecd.org/eco/growth/49421421.pdf. Accessed 7 Oct 2022.

[36] Kawachi I, Subramanian SV, Berkman LF, Kawachi I, GMM (2014). Income inequality. Social Epidemiology.

[37] Organisation for Economic Co-operation and Development. Education at a glance. 2022. http://www.oecd.org/education/education-at-a-glance-19991487.htm. Accessed 7 Oct 2022.

[38] College Board. Trends in Higher Education. 2022. https://trends.collegeboard.org/sites/default/files/2016-trends-college-pricing-web_0.pdf. Accessed 7 Oct 2022.

[39] World Health Organization (WHO). Achieving better oral health as part of the universal health coverage and noncommunicable disease agendas towards 2030. 2020. https://apps.who.int/gb/ebwha/pdf_files/EB148/B148_8-en.pdf. Accessed 30 Jul 2022.

[40] Currie C, Alemán-Díaz AY. The importance of large-scale (cross-national) data collection on early adolescents (10–15 years old): shedding light on socioeconomic and gender inequalities in health. 2015. https://www.unicef-irc.org/article/1157-the-importance-of-large-scale-cross-national-data-collection-on-early-adolescents.html. Accessed 20 May 2021.

[41] Roberts C, Freeman J, Samdal O, Schnohr CW, Looze ME, Nic Gabhainn S (2009). The Health Behaviour in School-aged Children (HBSC) study: Methodological developments and current tensions. Int J Public Health.

[42] World Health Organization (WHO). NCDs | Global school-based student health survey (GSHS). 2020. https://www.who.int/ncds/surveillance/gshs/datasets/en/. Accessed 20 May 2021.

[43] World Health Organization (WHO). Health Behaviour in School-aged Children (HBSC) data source - European Health Information Gateway. 2020. https://gateway.euro.who.int/en/datasets/hbsc/. Accessed 20 May 2021.

[44] United Nations Sustainable Development Solutions Network. Sustainable Development Report. 2021. https://dashboards.sdgindex.org/. Accessed 10 Jul 2022.

[45] Lana R, Nekkab N, Siqueira AM, Peterka C, Marchesini P, Lacerda M (2021). The top 1%: quantifying the unequal distribution of malaria in Brazil. Malar J.

[46] Abeles J, Conway DJ (2020). The Gini coefficient as a useful measure of malaria inequality among populations. Malar J.

[47] Kiadaliri AA, Saadat S, Shahnavazi H, Haghparast-Bidgoli H (2014). Overall, gender and social inequalities in suicide mortality in Iran, 2006–2010: A time trend province-level study. BMJ Open.

[48] Naghavi M, Wang H, Lozano R, Davis A, Liang X, Zhou M (2015). Global, regional, and national age-sex specific all-cause and cause-specific mortality for 240 causes of death, 1990–2013: A systematic analysis for the Global Burden of Disease Study 2013. Lancet.

[49] Steinbeis F, Gotham D, von Philipsborn P, Stratil JM (2019). Quantifying changes in global health inequality: the Gini and Slope Inequality Indices applied to the Global Burden of Disease data, 1990–2017. BMJ Glob Heal.

[50] UNICEF. National Gini index, 2003–2017. 2018. https://www.unicef.cn/en/figure-27-national-gini-index-20032017. Accessed 10 Jun 2022.

[51] Jargowsky PA. Ecological Fallacy. In: Encyclopedia of Social Measurement. Amsterdam: Elsevier; 2005. pp. 715–22.

[52] Moshagen M, Musch J, Ostapczuk M, Zhao Z (2010). Reducing socially desirable responses in epidemiologic surveys: an extension of the randomized-response technique. Epidemiology.

[53] Johnson TP, van de Vijver FJR. Social desirability in cross-cultural research. In: Harkness JA, van de Vijver FJR, Mohler PP, editors. Cross-Cultural Survey Methods. New York: Wiley; 2003. pp. 195–204.

[54] Gil GS, Morikava FS, Santin GC, Pintarelli TP, Fraiz FC, Ferreira FM (2015). Reliability of self-reported toothbrushing frequency as an indicator for the assessment of oral hygiene in epidemiological research on caries in adolescents: a cross-sectional study. BMC Med Res Methodol.

[55] Bernabé E, Sheiham A, Sabbah W (2009). Income, income inequality, dental caries and dental care levels: an ecological study in rich countries. Caries Res.

[56] Bernabé E, Marcenes W (2011). Income inequality and tooth loss in the United States. J Dent Res.

[57] Ting S, Zang W, Chen C, Chen D (2022). Income distribution and health: What do we know from Chinese data?. PLoS ONE.

[58] Kondo N, Sembajwe G, Kawachi I, Van Dam RM, Subramanian SV, Yamagata Z (2009). Income inequality, mortality, and self rated health: meta-analysis of multilevel studies. BMJ.

[59] Matthew P, Brodersen DM (2018). Income inequality and health outcomes in the United States: An empirical analysis. Soc Sci J.

[60] Sfm C, Van Cauwenberg J, Maenhout L, Cardon G, Lambert EV, Van Dyck D (2020). Inequality in physical activity, global trends by income inequality and gender in adults. Int J Behav Nutr Phys Act..

[61] MacKinnon DP, Krull JL, Lockwood CM (2000). Equivalence of the Mediation, Confounding and Suppression Effect. Prev Sci.

[62] Osypuk TL, Joshi P, Geronimo K, Acevedo-Garcia D (2014). Do Social and Economic Policies Influence Health? A Review. Curr Epidemiol reports.

[63] Singh K, Reddy KS, Prabhakaran D (2011). What are the Evidence Based Public Health Interventions for Prevention and Control of NCDs in Relation to India?. Indian J Community Med.

